# Cognitive Control in Adolescence: Neural Underpinnings and Relation to Self-Report Behaviors

**DOI:** 10.1371/journal.pone.0021598

**Published:** 2011-06-28

**Authors:** Jessica R. Andrews-Hanna, Kristen L. Mackiewicz Seghete, Eric D. Claus, Gregory C. Burgess, Luka Ruzic, Marie T. Banich

**Affiliations:** 1 The Institute of Cognitive Science, University of Colorado at Boulder, Boulder, Colorado, United States of America; 2 The Mind Research Network, Albuquerque, New Mexico, United States of America; 3 Department of Psychology and Neuroscience, University of Colorado at Boulder, Boulder, Colorado, United States of America; 4 Department of Psychiatry, University of Colorado at Denver, Denver, Colorado, United States of America; University College London, United Kingdom

## Abstract

**Background:**

Adolescence is commonly characterized by impulsivity, poor decision-making, and lack of foresight. However, the developmental neural underpinnings of these characteristics are not well established.

**Methodology/Principal Findings:**

To test the hypothesis that these adolescent behaviors are linked to under-developed proactive control mechanisms, the present study employed a hybrid block/event-related functional Magnetic Resonance Imaging (fMRI) Stroop paradigm combined with self-report questionnaires in a large sample of adolescents and adults, ranging in age from 14 to 25. Compared to adults, adolescents under-activated a set of brain regions implicated in proactive top-down control across task blocks comprised of difficult and easy trials. Moreover, the magnitude of lateral prefrontal activity in adolescents predicted self-report measures of impulse control, foresight, and resistance to peer pressure. Consistent with reactive compensatory mechanisms to reduced proactive control, older adolescents exhibited elevated transient activity in regions implicated in response-related interference resolution.

**Conclusions/Significance:**

Collectively, these results suggest that maturation of cognitive control may be partly mediated by earlier development of neural systems supporting reactive control and delayed development of systems supporting proactive control. Importantly, the development of these mechanisms is associated with cognitive control in real-life behaviors.

## Introduction

Recent advances in neuroimaging research have increased our understanding of the development and decline of cognitive ability across the human lifespan. Despite such progress, the neural underpinnings of transition periods within the lifespan are less well-established. Adolescence represents a neurobiological transition period sometimes marked by impulsivity, lack of foresight, poor decision-making, elevated emotional reactivity, and sensation-seeking behavior [reviewed in 1], [2], [3]. Some of these behavioral characteristics have been attributed to deficits in cognitive control, which has been described as the ability to “override or augment reflexive and habitual reactions in order to orchestrate behavior in accord with [one's] intentions” [4, p 59].

Recent accounts of cognitive control suggest it can be differentiated into multiple component processes. In the “Cascade-of-Control Model,” we recently distinguished between processes related to the implementation and maintenance of a top-down attentional set from those related to later stages of selection through response selection and evaluation [5], [6], [7]. Lateral prefrontal regions including the posterior dorsolateral prefrontal cortex (pDLPFC) are thought to proactively bias attention towards task-relevant goals and representations [8], while medial prefrontal regions such as the anterior cingulate cortex (ACC) may support more reactive aspects of attentional control, especially late-stage processing including response-related and evaluative aspects of control [9]. Similarly, Braver and colleagues' “Dual Mechanisms of Control Model” refers to “proactive” control as preparatory processes sometimes sustained over the course of the task, and “reactive” control as transient control processes implemented following perception of the stimulus [10].

Of relevance to lifespan development, older adults appear to shift from a proactive strategy to a reactive strategy, likely mediated by declining dopamine availability and compromised lateral prefrontal function [11], [12]. On the other end of the spectrum, young children (age 3) experience difficulties utilizing predictive information and maintaining that information over a few seconds, while older children (age 8) do so more readily [13].

A growing number of studies suggest that adolescents exhibit different patterns of functional activation than adults during tasks requiring cognitive control [14]–[26], [ reviewed in 3], [27]–[30]. However, the precise regions that exhibit group differences and the nature of those differences reported in prior literature are somewhat inconsistent, perhaps due to the use of varied tasks, fMRI designs and analysis (i.e. blocked vs. event-related), and group differences in performance across studies. Nevertheless, considering the continued structural and functional maturation of the prefrontal cortex across adolescence [reviewed in 2], [27]–[34], one important question is whether there is on-going development of the neural structures that allow one to proactively implement and maintain a task set.

The present study aimed to extend previous adolescent neuroimaging findings by examining the developmental trajectories of both proactive and reactive control and by exploring the neural predictors of individual differences in real-world measures of cognitive function [see 35,36]. To examine the neural structures supporting proactive and reactive aspects of cognitive control, a large sample of adolescents and adults performed a “hybrid” blocked/event-related version of the Color-Word Stroop Task [37] – a classic test of executive function. This hybrid design allowed estimates of more sustained activation averaged across blocks of easy and difficult trials and more transient differences between trial types within a block. In addition, to examine individual differences in real-world behaviors across development, we administered self-report measures of impulsivity, lack of foresight, and susceptibility to peer pressure.

We predicted that functional maturation of sustained, proactive aspects of control would continue throughout adolescence, and that adolescents might compensate with increased reliance on late-stage reactive mechanisms, particularly for difficult trials requiring response-related interference resolution. As suggested by prior models, immature proactive control might manifest as decreased sustained blood-oxygenated level-dependent (BOLD) activity in lateral prefrontal regions including pDLPFC, whereas reactive response-related compensatory mechanisms might manifest as elevated transient activity in regions such as the ACC and the supplementary motor area (SMA) [5]. Furthermore, if lateral prefrontal regions play a role in proactive top-down control, one might expect that adolescents with increased sustained activity in these regions would report a better ability to control their behavior and plan ahead.

## Materials and Methods

### Participants

Participants under the age of 18 were recruited from after-school programs, community centers, and through fliers on local bus routes in metropolitan Denver. Adult participants were recruited via flyers in communities similar to those where youth were recruited, including bulletin boards for custodial and maintenance staff at hospitals and at community colleges, grocery stores, churches, and local bus routes. Advertisements were also placed in local newspapers and email lists. Before study enrollment, participants were screened to exclude those who were left-handed, those who learned English as a non-native language, those who were pregnant or trying to become pregnant, and those with a history of psychiatric or neurological disorders, drug abuse, head trauma, claustrophobia, metallic implants or other MRI contraindications. Informed consent was obtained in writing from all participants of age to provide consent (≥18), and from the legal guardians or parents of all participants below the age of consent (<18). Additionally, all minors signed an additional “assent” form explaining the procedures of the study. All procedures were approved by the Colorado Multiple Institutional Review Board and participants were reimbursed with cash.

A total of 41 adolescent individuals (ages 14–17) and 43 adult individuals (ages 18–25) met the above criteria and participated in the study. However, 7 adolescent and 7 adult participants either failed to complete the study or produced unusable imaging data due to scanner artifacts and/or excessive movement (>2 mm linear displacement). Additionally, 1 adolescent and 2 adults were excluded for failing to respond on a considerable number of trials, while 1 adolescent and 1 adult qualified as outliers for their respective groups (>2.5 SD from the group mean) on overall accuracy and were excluded from subsequent analyses. No participants were considered outliers on overall response time (RT).

After eliminating participants based on the above criteria, 32 adolescents (15.6 yr, 14–17, 53.1% male) and 33 adults (21.9 yr, 18–25, 45.5% male) yielded useable imaging and behavioral data and were analyzed in the present manuscript. Demographic information is outlined in **Table 1**. Additionally, participants were administered a written two-subtest version of the Wechsler Abbreviated Scale of Intelligence (WASI; Psychological Corporation, 1999), which has been previously normed for use by participants aged 6–89. The two-subtest version includes the Vocabulary subtest and a Matrix Reasoning subtest. Scores from the separate subtests were combined into a full-scale IQ (FSIQ) measure. As outlined in **Table 1**, adolescent and adult groups were matched on gender and parental education, but adults exhibited significantly higher FSIQ scores compared to adolescents (*t* = 2.33, *p* = 0.022).

**Table 1:** Participant demographics.

	Adolescents	Adults
N	32	33
Mean Age (yr)	15.6	21.9
Age Range (yr)	14–17	18–25
% Males	53.1	45.5
Fullscale IQ	100.2 (9.54)	106.8 (12.51)*
Mother's Education	12.9 (1.61)	13.2 (1.14)
Father's Education	13.2 (1.49)	13.5 (1.26)

*t = 2.33, *p* = 0.022.

### Task Paradigm

Participants completed a hybrid blocked/event-related version of the Stroop Color-Word task [37]. This paradigm has been shown to be particularly sensitive to individual differences within a population as well as distinguishing control from clinical groups [38], [39]. In addition to fixation trials, three trial types were included in the task paradigm: congruent, incongruent, and neutral. On congruent trials, the color of the ink was consistent with the semantic content of the word (e.g. “red” in red ink). On incongruent trials, the ink color and word meaning were inconsistent (e.g. “red” in green ink). On neutral trials, a non-color word was presented in a colored font (e.g. “bond” in blue ink). Neutral words were matched with incongruent and congruent words for word length. Participants were instructed to identify the ink color of each word using one of four buttons on button boxes held in his/her left and right hands. On each trial, the word appeared for 1500 ms, followed by 500 ms of fixation between trials.

Participants completed three Stroop task runs while scanned with fMRI. Each run comprised four, 24 s fixation (F) blocks interleaved with nine, 24 s task blocks. Three task blocks were grouped into triads and each triad consisted of a congruent (C), incongruent (I), and neutral (N) block, first-order counterbalanced across triads and participants. For example, the order of one run might be F-CIN-F-INC-F-NCI-F. Half of the trials in each block consisted of stimuli that were specific to that block (i.e. incongruent (i), congruent (c), neutral (n)) and the remaining half of the trials consisted of neutral stimuli that appeared across all blocks. The trial types within blocks were pseudo-randomly ordered such that no more than two trials of the same type could appear in a row. The inclusion of block-general neutral words within each block allows one to examine transient changes in attentional control (i.e., within a block). In addition, the presence of these neutral words minimizes any potential habituation effects that might occur in the incongruent and congruent blocks and ensures that within the congruent block participants do not “cheat” and adopt a strategy of reading the word. Hence, within the congruent blocks, six congruent trials (c) were mixed with six block-general neutral trials (n_c_) to allow for comparisons between trial types within blocks. Similarly, within incongruent blocks, six incongruent trials (i) were mixed with six block-general neutral trials (n_i_). Neutral blocks consisted of 12 block-general neutral trials (n_n_) and 12 neutral trials that were specific to the neutral block (n). In total, participants completed 324 task trials, with 54 trials corresponding to each trial type.

### Self-Report Questionnaires

Outside the MRI scanner, participants completed a variety of self-report questionnaires as part of a larger, ongoing study. These questionnaires asked participants to rate the degree to which several cognitive, social, and emotional characteristics were accurate representations of their own personalities and behaviors. All questionnaires were completed on a computer in a quiet testing room.

The *Weinberger Adjustment Inventory* [WAI; 40] was administered to assess the degree to which participants were able to control their impulses and suppress aggressive behaviors. The Impulse Control subscale, in which we were most interested, was comprised of 8 items (e.g. “I do things without giving them enough thought”). Participants rated the self-descriptiveness of these items using a 1–5 Likert scale (1 = false, 2 = somewhat false, 3 = not sure, 4 = somewhat true, 5 = true), with some items being reverse scored. The suppression of aggression subscale consisted of 7 items (e.g. “If someone tries to hurt me, I make sure I get even with them”) to which participants responded using the same scale.

Participants also completed the *Future Orientation Questionnaire* [41], a 15-item questionnaire that assesses the degree to which individuals plan ahead, anticipate future consequences, and think about the future. Items consisted of two opposing statements separated by the word “BUT.” Participants selected the statement that they believe best fit their own personality. They then quantified whether the chosen statement was “very true” or “sort of true.” The 15-items were divided into three separate, 5-item subscales: *Planning Ahead* (e.g. “Some people think that planning things out in advance takes all the fun out of things” BUT “Other people think that things work out better if they are planned out in advance”), *Anticipation of Future Consequences* (e.g. “Some people have trouble imagining how things might play out over time” BUT “Other people are usually pretty good at seeing in advance how one thing can lead to another”), and *Time Perspective* (e.g. “Some people would rather be happy today than take their chances on what the future may bring” BUT “Other people will give up their happiness now so that they can get what they want in the future”). Scores ranged from 1–4, where 1 = least future orientation and 4 = most future orientation.

The 10-item *Resistance to Peer Influence* questionnaire [42] was administered to assess cognitive control, particularly in social situations. Similar to the *Future Orientation* questionnaire, participants selected one of two opposing statements that they believe best fit their own personality (e.g. “Some people would do something that they knew was wrong just to stay on their friends' good side” BUT “Other people would not do something they knew was wrong just to say on their friends' good side”). Next, participants quantified whether the chosen statement was “very true” or “sort of true.” Scores ranged from 1–4 where 1 = least resistance to peer influence and 4 = most resistance to peer influence.

Finally, participants completed the 19-item *Sensation Seeking Scale* [43]. Seven of the 19 items assessed the degree to which participants engage in unplanned and impulsive behaviors (e.g. “I hardly ever spend much time on the details of planning ahead”). Since we were interested in these questions for the purpose of the present study, we averaged the seven items into an *Impulsivity and Lack of Foresight* subscale. The 12 remaining items, which were not of interest for the present study, generally assessed the degree to which participants engage in thrill-seeking behavior (e.g. “I like doing things just for the thrill of it”). Participants indicated whether each item was “true” or “false.” Higher scores indicate greater impulsivity/lack of foresight and thrill-seeking behavior.

Because planning and foresight (which are often negatively correlated with impulsivity) [44], represent key characteristics of “proactive” control, we hypothesized that sustained patterns of prefrontal activity during the Stroop Task would predict self-report measures of planning ability and impulse control, particularly in the adolescent group. Additionally, we reasoned that the neural underpinnings of proactive control might extend to the social domain, particularly the ability to resist the influence of peer pressure. Prior studies have found positive relationships between resistance to peer influence and impulse control in adolescents [45], as well as positive relationships with the strength of task-related functional connectivity between prefrontal cortex and regions involved in action observation [35].

To examine these possibilities, we selected scores from the *Weinberger Adjustment Inventory Impulse Control* subscale, the *Planning Ahead* subscale of the *Future Orientation* questionnaire, the *Impulsivity/Lack of Foresight* subscale of the *Sensation Seeking Scale*, and the *Resistance to Peer Influence* questionnaire. Principal Components Factor Analyses on these measures (after controlling for the effect of age) resulted in a single significant factor explaining 52% of the variance in participants' scores (eigenvalue = 2.09). However, while the measures relating to impulse control and planning ability loaded heavily on the single factor (factor loadings for all three measures >0.75), “Resistance to Peer Influence” loaded on this factor to a smaller degree (factor loading = 0.30). These results suggest that the control of social behavior may be governed somewhat by other factors.

Based on the extraction of a single significant factor, we converted each self-report measure to z-scores separately for each group (i.e. adolescents, adults), reverse-scored the *Impulsivity/Lack of Foresight* measure, and averaged the z-scores across measures to create a cognitive/social control composite. Higher scores are indicative of greater cognitive/social control. Then, for purposes of comparing scores across adolescent and adult groups, we computed z-scores using the mean of the entire group.

### MRI Data Acquisition

Scanning was performed on a 3T GE Signa scanner (Milwaukee, WI), with a standard 4-channel head coil. Three-dimensional, high resolution, T1-weighted IR-SPGR anatomical images were acquired using the following parameters: repetition time (TR) = 9.61 ms, echo time (TE) = 2.0 ms, inversion time (TI) = 500 ms, field-of-view (FOV) = 220 mm, matrix size = 256×256, in-plane resolution = 0.87 mm×0.87 mm, slice thickness = 1.7 mm, 124 coronal slices. Additionally, T2*-weighted gradient echo, echo-planar functional images (with ramp sampling) were acquired using the following parameters: TR = 2000 ms, TE = 32 ms, flip angle = 77°, FOV = 220 mm, matrix size = 64×64, in-plane resolution = 3.44 mm×3.44 mm, slice thickness = 4 mm, 29 interleaved slices acquired parallel to the AC-PC line, 163 volumes.

Stimuli were programmed using E-Prime software (Psychology Software Tools, Inc) and were viewed through MRI-compatible goggles. Participants were given earplugs to dampen scanner noise and an air pillow was inflated around each participant's head to minimize head movement. Participants held a four-button fiber-optic button box in each hand and responded to each trial with one of two buttons per hand colored in either red, green, blue, or yellow ink.

### Data Processing and Statistical Analysis

#### fMRI Preprocessing

To prepare the data for statistical analyses, a series of image preprocessing steps were performed using FSL tools (FMRIB, Oxford, UK, www.fmrib.ox.ac.uk). The first 7 volumes were discarded to ensure scanner intensity stabilization, followed by motion correction using a rigid-body translation and rotation algorithm (MCFLIRT) and extraction of brain tissue (BET). Next, the three functional runs were concatenated. Within FMRIB Easy Analysis Tool (FEAT), the 4D concatenated images were corrected for differences in slice timing, were spatially smoothed using an 8 mm full-width half-maximum (FWHM) Gaussian kernel, and were pre-whitened with FMRIB's Improved Linear Model (FILM). Run constants and linear trends were modeled within each GLM to account for differences in overall intensity between runs and to remove low-frequency scanner drift.

#### Modeling Blocked Effects

As described previously, the hybrid block/event-related task paradigm was designed such that block effects and event-related effects would be modeled within separate GLMs. To examine block-effects, three separate regressors (one for each block type: congruent (C), incongruent (I), neutral (N)) were modeled by convolving a double-gamma response function with the onsets of each initial correct trial in a string of correct trials as an epoch. Additionally, three separate regressors were modeled to account for incorrect (error) trials within each block type. In order to ensure that blocked effects were independent of these error trials, each blocked regressor was orthogonalized with respect to the corresponding error regressor. As described in more detail in the results, contrasts of interest include each of the three block types compared to fixation (i.e. C-F, I-F, N-F), I-N (i.e. the Stroop interference effect), C-N (i.e. the Stroop facilitation effect), and I-C (i.e., a measure of cognitive control in the face of conflict).

#### Modeling Event-Related Effects

To explore event-related effects, seven regressors corresponding to separate trial types were modeled in a single GLM: incongruent trials (i), neutral trials within incongruent blocks (n_i_), congruent trials (c), neutral trials within congruent blocks (n_c_), neutral trials (n), neutral trials within neutral blocks (n_n_), and incorrect trials (e). For each regressor, a double-gamma response function was convolved with the onset of each trial. The contrast of i - n_i_ trials (i.e. the trial-related Stroop interference effect) was explored for the purposes of the present study since this contrast reflects the greatest difference in transient demand on executive control.

#### Statistical Analyses

FMRIB's Improved Linear Model (FILM) was used to separately compute the blocked and event-related GLMs for individual participants. Each participant's data was registered to the Montreal Neurological Institute (MNI) stereotaxic template using FMRIB's Linear Image Registration Tool (FLIRT) two-step process. Higher-level, group analyses for each contrast of interest (i.e. I block parameter estimate – N block parameter estimate) were computed using FMRIB's Local Analysis of Mixed Effects (FLAME), which models the within-subject variance using fixed-effects analyses and the between-subject variances using random-effects analyses. Within these higher-level GLMs, covariates of non-interest included each participant's fullscale IQ, overall error rate, and overall non-response rate. Thus, one can be reasonably confident that observed effects of age are not driven by individual differences in IQ or error rate. Within FLAME, group difference analyses (i.e. adults vs. adolescents; 16–17 yr olds vs. 14–15 yr olds) for each contrast of interest were computed using two-sample *t*-tests.

Higher-level whole-brain correlation analyses between fMRI Stroop interference estimates (parameter estimates for the contrasts of I-N blocks and i-n_i_ trials) and composite scores reflecting self-report measures of cognitive/social control were performed using FSL's robust regression to minimize the impact of outliers [46]. Whole-brain correlation analyses were performed separately for the adolescent and adult groups, and age was entered as a covariate of non-interest in each GLM.

To determine appropriate voxel-wise and cluster-wise statistical thresholds for functional images, Monte Carlo simulations were performed using the AlphaSim algorithm [47]. As demonstrated by the algorithm, clusters of activation were considered significant if they exceeded a voxel-wise threshold of *p*<0.005 (two-tailed) and a cluster size of 103 contiguous voxels. The peak x,y,z coordinate in MNI space was extracted from each significant cluster and listed in fMRI tables, as well as the number of voxels comprising each cluster and the z-statistic corresponding to the adolescent, adult, and group difference maps separately. In some cases, a significant cluster comprised a large number of voxels and spanned distant brain regions. In such cases, the larger cluster was subjected to increasingly stricter voxel-wise thresholds and increasingly smaller cluster-wise thresholds (in accordance with AlphaSim) until it partitioned into smaller clusters. The peak coordinates from these smaller clusters are listed in the table. The anatomical description of each significant cluster was classified primarily using the Harvard-Oxford Probabilistic Structural Atlas. If a smaller cluster spanned multiple regions, all regions are listed (e.g. IFG/MFG). We omit the reporting of Brodmann areas in statistical tables since Brodmann areas can vary substantially across atlases and are often determined from a single individual as opposed to probabilistic brain template [see 48].

Behavioral data reported in the results and **Table 2** was calculated after removing subjects that fell above or below 2.5 SD of the group mean. Unless otherwise-noted, the significance of statistical tests (e.g. paired *t*-tests, independent samples *t*-tests, correlation analyses) were calculated using two-tails.

**Table 2:** Behavioral performance.

	Abbreviation	Adolescents	Adults	*t*
		Mean (SD)	Mean (SD)	
**Accuracy**				
*Incongruent Blocks*	I	*0.94 (0.05)*	*0.96 (0.03)*	*1.22*
Incongruent trials	i	0.93 (0.06)	0.93 (0.06)	0.25
Neutral trials in I blocks	n_i_	0.97 (0.03)	0.98 (0.03)	0.72
Proportion trial interference		−0.04 (0.04)	−0.05 (0.06)	−0.87
*Congruent Blocks*	*C*	*0.97 (0.02)*	*0.98 (0.01)*	*1.76*
Congruent trials	c	0.96 (0.03)	0.97 (0.02)	1.34
Neutral trials in C blocks	n_c_	0.97 (0.02)	0.98 (0.01)	1.92
Trial facilitation		−0.01 (0.04)	−0.01 (0.03)	−0.09
*Neutral Blocks*	*N*	*0.98 (0.02)*	*0.98 (0.02)*	*0.78*
Neutral trials	n	0.98 (0.02)	0.98 (0.02)	0.52
Neutral trials in N blocks	n_n_	0.97 (0.02)	0.98 (0.02)	0.85
*Proportion Blocked Interference*		−0.02 (0.04)	−0.02 (0.03)	−0.17
*Proportion Blocked Facilitation*		−0.01 (0.02)	0.01 (0.02)	0.70
**Response Time**				
*Incongruent Blocks*	I	*775 (122)*	*769 (97)*	*−0.18*
Incongruent trials	i	838 (140)	840 (119)	0.06
Neutral trials in I blocks	n_i_	712 (114)	699 (91)	−0.51
Proportion trial interference		0.18 (0.11)	0.20 (0.12)	0.54
*Congruent Blocks*	*C*	*701 (101)*	*681 (88)*	*−0.82*
Congruent trials	c	706 (107)	673 (79)	−1.43
Neutral trials in C blocks	n_c_	695 (99)	673 (75)	−0.97
Trial facilitation		0.02 (0.05)	0.00 (0.05)	−1.71
*Neutral Blocks*	*N*	*697 (100)*	*683 (79)*	*−0.62*
Neutral trials	n	706 (101)	687 (80)	−0.85
Neutral trials in N blocks	n_n_	688 (102)	672 (70)	−0.74
*Proportion Blocked Interference*		0.11 (0.09)	0.12 (0.07)	0.44
*Proportion Blocked Facilitation*		0.00 (0.04)	0.00 (0.04)	−0.88

*Note:* Group statistics represent the comparison of Adults>Adolescents. No group comparisons were significant at *p*<0.05. For each variable, outliers >2.5 SD from each group's mean were excluded for calculation purposes.

Proportion Trial Interference = (i−n_i_)/n_i_; Proportion Trial Facilitation = (c−n_c_)/n_c_.

Proportion Blocked Interference = (I−N)/N; Proportion Blocked Facilitation = (C−N)/N.

### Interpretation of Blocked and Event-Related Results

Note that our design is slightly different from “state-item” designs, which ensure that blocked and event-related effects are statistically independent of each other because both effects are modeled within a single GLM [49]–[51]. In such state-item designs, blocked effects are considered estimates of “sustained” activity that persist over the length of the block, while event-related effects are considered estimates of “transient” activity in response to single trials. However, the jittered fixation trials required by state-item designs necessitate a longer scanning duration, which might be difficult for younger populations. Additionally, these fixation trials might interfere with maintenance of a top-down attentional set since they are linked to activation of the default network [52]. Therefore, we adopted a slightly different design whereby task blocks consisted of 12 back-to-back task trials.

In our current hybrid design, it is important to note that event-related contrasts are pure measures of “transient” differences in activity since different event types are compared within blocks. Therefore, differences between events are independent of the level of sustained activation across the block. However, blocked effects are affected by both sustained and transient activation [see 53 for further discussion]. In consideration of this issue, we reasoned that regions showing group differences in blocked effects in the absence of group differences in event-related effects were likely a result of sustained rather than transient activity. As demonstrated in the results, we did not observe any significant differences in event-related activity between adults and adolescents on a whole-brain level.

To further examine the possibility that blocked effects were confounded by transient effects, we treated areas that yielded significant adult>adolescent group differences in blocked activity as ROIs and tested whether they yielded significant differences in transient activation. None of these regions exhibited a significant group difference in event-related BOLD activity, even at a low threshold of *p*<0.05. For this reason, we consider blocked group differences to be driven primarily by group differences in “sustained” activity and event group differences to be driven by group differences in “transient” activity.

## Results

### Behavioral Results

Consistent with prior studies examining the Stroop color-word task [reviewed in 54], response time and accuracy significantly differed across conditions, both when examined in a blocked and trial-by-trial fashion. As would be expected, the two groups demonstrated robust Stroop interference effects. Participants were significantly slower and less accurate when responding to incongruent blocks (I) than to neutral blocks (N) (*RT Effects:* paired *t*-test, Adolescents: *t*(31) = 7.44, *p*<0.001, Adults: *t*(32) = 9.82, *p*<0.001; *Accuracy Effects:* paired *t*-test, Adolescents: *t*(30) = −2.91, *p*<0.01, Adults: *t*(31) = −4.12, *p*<0.001). Additionally, participants were significantly slower and less accurate when responding to incongruent trials (i) than to neutral trials within incongruent blocks (n_i_) (*RT Effects:* paired t-test, Adolescents: *t*(31) = 9.59, *p*<0.001, Adults: *t*(32) = 9.31, *p*<0.001; *Accuracy Effects:* paired *t*-test, Adolescents: *t*(29) = −4.85, *p*<0.001, Adults: *t*(31) = −4.88, *p*<0.001).

No group differences in accuracy or RT were observed (**Table 2**). Furthermore, when group status was ignored and age was instead coded as a continuous variable, age did not exhibit either a linear effect on behavior (Blocked RT interference: *r*(62) = 0.066, *p* = 0.61; Trial RT interference: *r*(62) = 0.046, *p* = 0.72; Blocked accuracy interference: *r*(62) = 0.15, *p* = 0.24; Trial accuracy interference: *r*(60) = 0.095, *p* = 0.46) or a quadratic effect on behavior (Blocked RT interference: *F*(61) = 0.66, *p* = 0.52; Trial RT interference: *F*(61) = 1.98, *p* = 0.15; Blocked accuracy interference: *F*(61) = 0.77, *p* = 0.47; Trial accuracy interference: *F*(59) = 1.61, *p* = 0.21). Additionally, age did not exhibit a significant linear effect on behavior within the adolescent group alone (Blocked RT interference: *r*(30) = 0.13, *p* = 0.47; Trial RT interference: *r*(30) = 0.27, *p* = 0.13; Blocked accuracy interference: *r*(29) = 0.28, *p* = 0.13; Trial accuracy interference: *r*(29) = 0.22, *p* = 0.23).

However, significant relationships between RT interference (but not accuracy interference) and full-scale IQ were observed such that, across the two groups, individuals with higher FSIQ exhibited larger RT interference effects (FSIQ×Blocked RT interference: *r*(61) = 0.26, *p* = 0.039; FSIQ×Trial RT interference: *r*(61) = 0.43, *p* = 0.001). As mentioned in the **Materials and Methods**, adults exhibited overall higher FSIQ than adolescents (**Table 1**). In an attempt to minimize the effect of group differences in FSIQ on our higher-level imaging results in all statistics where group was a factor, we included, as a covariate of non-interest, the portion of FSIQ that was not shared with Stroop RT interference. For the blocked fMRI GLM, we included the residual from the linear regression between FSIQ and proportion blocked RT interference (described above). Likewise, for the event-related GLM, we included the residual from the linear regression between FSIQ and proportion trial RT interference. Importantly, inclusion of these covariates did not change the overall pattern of observed results, and when we restricted our analyses to a subset of 28 adolescents and 25 adults who were matched on FSIQ (adolescents: 102.5, adults: 103.0), we observed similar group effects.

### Imaging Results

#### Blocked Analyses

*Conditions vs. Fixation:* Although task blocks vary in their demands for cognitive control, all blocks encourage sustained, top-down biasing of attention towards task-relevant goals (color identification) and away from the more automatic task-irrelevant processes (word reading). Even in the neutral condition where control demands are lessened compared to incongruent and congruent trials, the presence of a word compared to non-word strings interferes with the process of color naming [55]. Thus, one might expect that under-developed neural mechanisms for maintaining a proactive, top-down attentional set would manifest itself during all block types compared to fixation.

To examine the age-related neural underpinnings associated with proactive maintenance of task goals, we first performed a contrast of task blocks versus fixation baseline blocks separately for each block type (I, C, N) and group (adolescents, adults). For each of the three contrasts, both groups activated several frontal and parietal brain regions implicated in top-down control, including regions at or near the middle and inferior frontal gyrus, ACC, anterior inferior parietal cortex, superior parietal cortex, and precuneus. Importantly, consistent with the possibility that adolescents may be less effective at maintaining a proactive attentional set, whole-brain analyses performed at the group level revealed increased activity in adults compared to adolescents during all task blocks, notably in clusters corresponding approximately to mid and posterior dLPFC, extending into inferior frontal junction (IFJ) (**Figure S1**; **Table S1**). The superior parietal lobule, a region implicated in selective attention [56], was also activated more for adults compared to adolescents across all task blocks.

*Differences between Conditions:* Our next objective was to examine group differences in activation between blocks. The contrast between I and N blocks isolates a number of control processes: a) increased demands to bias attention towards task-relevant processes (color information) and away from task-irrelevant processes (word reading), b) the requirement to distinguish between two sources of color information that compete for attentional priority (color information extracted from the ink color and color information extracted from word reading) as compared to just one (on neutral trials color information is only contained in the ink color), and c) the requirement to distinguish between conflicting semantic and stimulus-response mappings (color information extracted from word reading has a different meaning and leads to a different response than color information extracted from the ink color).

**Figure 1:**
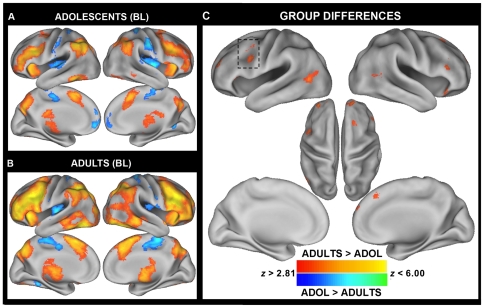
Between-group differences in blocked Stroop activity reveal adolescents under-activate lateral prefrontal cortex compared to adults. Significant clusters of activation for the blocked Stroop contrast of Incongruent blocks - Neutral blocks are displayed separately for **A.** Adolescents and **B.** Adults. Voxels in red indicate greater activity for I blocks compared to N blocks and voxels in blue indicate greater activity for N blocks compared to I blocks. While both groups of participants activated a network of fronto-parietal regions implicated in cognitive control, **C.** adults exhibited significantly greater activity in lateral prefrontal regions, dorsal medial prefrontal cortex, and temporal-occipital regions. *Note:* A voxel-wise threshold of *p*<0.005 and a cluster-wise threshold of >103 contiguous voxels were applied to the statistical maps using Monte Carlo permutation simulations (AlphaSim). Results are projected onto a surface template (Caret Software) [83].

**Figure 2:**
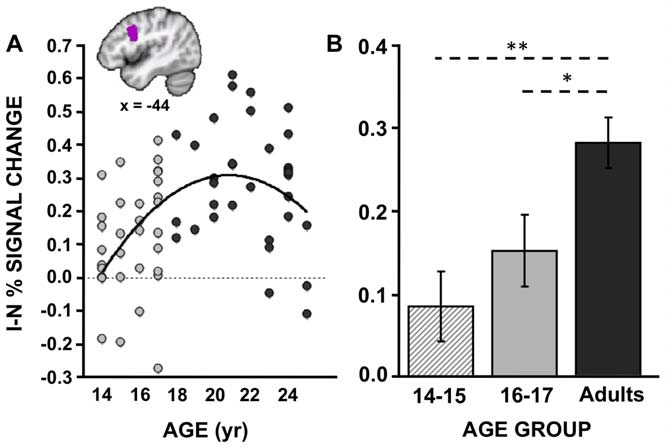
Relationships between blocked Stroop activity and age. Percent signal change for the contrast of Incongruent (I) - Neutral (N) blocks was extracted from the lateral prefrontal cluster highlighted in the box in Figure 1C. This region corresponds approximately to IFJ/pDLPFC and is shown in a sagittal slice in panel **A**, where the percent signal change within this region is plotted across age, yielding an inverted J-shaped function. **B.** The magnitude of activity in the same IFJ/pDLPFC region is plotted for the two adolescent age groups as well as the adult group. *Note:* *p<0.05; **p<0.01. Error bars reflect standard error of the mean.

While both groups exhibited increased activity on I blocks compared to N blocks in a network of frontal-parietal regions implicated in cognitive control (**Fig. 1A and 1B**), adults activated a number of primarily prefrontal regions to a greater degree than adolescents including those near the left IFJ/pDLPFC, bilateral anterior PFC/frontal pole, right inferior frontal gyrus (IFG)/anterior insula (aI), and medial PFC near BA8 (**Fig. 1C**; **Table 3**). To explore the relationship between percent signal change and age in these regions, we extracted, for each participant, the mean percent signal change for all voxels (exceeding a threshold of *p*<0.005) within an 8 mm radius sphere from each cluster's peak. Post-hoc analyses revealed that though activity in many of the regions exhibited significant linear relationships with age, the relationship tended to be best fit by a quadratic function, with activation slowly increasing until approximately age 21 and decreasing thereafter (**Table 3**). This inverted J-shaped relationship is clearly illustrated in the left IFJ/pDLPFC, a region which we predicted to exhibit group differences in blocked activation (see **Figure 2A**). These results suggest that, compared to adults, adolescents are less likely to proactively up-regulate top-down attentional resources during the more difficult incongruent blocks. However, though the slight decline in activity at higher ages might reflect increased efficiency at maintaining a top-down attentional set, it could also be explained by a shift in strategy use or alterations in underlying neural tissue. Future studies will be needed to further explore these alternative possibilities.

**Table 3:** fMRI Blocked Group Differences between Conditions.

	# Voxels	x	y	z	Peak *z*-statistic			% Variance Expl	
					Diff	Adults	Adol	linear	quad
**Incongruent – Neutral Blocks (Adults>Adol)**									
Frontal Pole (L)	213	−32	50	24	4.06	5.33	0.14	3.8	5.6
IFG/Middle Frontal Gyrus (R)	138	50	30	16	3.55	8.78	3.26	3.2	15.5**
Frontal Pole (R)	144	20	58	26	3.49	4.79	−0.19	2.3	3.5
Orbitofrontal Cortex (R)	109	38	26	−14	3.45	5.51	1.13	7.1	18.3**
PreCG/Middle Frontal Gyrus/IFG (L)	207	−44	6	30	3.44	8.28	4.02	12.1	23.4***
Frontal Operculum/Insula/IFG (L)	108	−42	22	2	3.44	5.33	0.71	2.6	6.4
Superior Frontal Gyrus/Paracingulate (R)	161	12	32	42	3.43	2.85	−2.53	9.0	12.4
Lateral Occipital Complex /MTG/Angular Gyrus (L)	270	−56	−64	8	3.74	3.52	−2.36	5.3	5.3
MTG/Angular Gyrus/Lateral Occipital Complex (R)	174	60	−52	12	3.50	4.55	−0.70	6.0*	8.7
**Incongruent – Congruent Blocks (Adults>Adol)**									
Frontal Pole (R)	201	24	60	22	3.87	4.51	−1.09	1.1	1.4
Middle Frontal Gyrus (R)	193	38	12	46	3.64	2.74	−2.82	20.8***	25.6***
Orbitofrontal Cortex (R)	172	42	26	−10	3.42	5.24	0.88	22.1***	28.3***
MTG/Angular Gyrus (R) *λ*		58	−54	8	4.41	3.07	−3.56	19.6***	23.8***
MTG/Superior Temporal Gyrus (R) *λ*		64	−38	−6	3.42	2.65	−2.75	4.5	5.6
MTG/Lateral Occipital Complex/SMG (L)	349	−52	−62	6	4.10	3.85	−2.58	10.0**	10.2*
Amygdala (R)	137	34	−2	−22	3.57	2.20	−3.15	10.1**	12.2*
Lingual Gyrus (R)	104	14	−60	−4	3.53	1.67	−3.49	2.9	3.5

*Note:* See Methods for details concerning region identification. No regions exhibited significantly greater activity in adolescents compared to adults. IFG = Inferior Frontal Gyrus; MTG = Middle Temporal Gyrus; SMG = Supramarginal Gyrus; STG = Superior Temporal Gyrus.

*λ* = regions part of a single cluster of 1335 voxels.

*p<0.05;

**p<0.01;

***p<0.001.

The contrast between I and C blocks isolates those attentional demands that are specific to conflicting semantic and response-related processes (described as “c” above). As demonstrated in **Table 3**, adults exhibited significantly increased activity within right prefrontal regions including the superior frontal gyrus/pDLPFC and aI/IFG. Additionally, group differences were observed in bilateral temporal-occipital cortex near middle temporal gyrus and the amygdala, arising because adolescents showed increased activity during congruent blocks, whereas adults showed increased activity during incongruent blocks. Many of the regions that exhibited group differences exhibited significant linear and quadratic relationships with age (**Table 3**).

Finally, Stroop facilitation effects were explored with the contrast of C versus N blocks. However, no significant group differences were observed.

#### Individual-Trial Analyses

Thus far, these results suggest that compared to adults, adolescents under-recruit a number of lateral prefrontal regions commonly implicated in proactive, sustained top-down control. Our next objective was to explore whether adolescents instead mainly rely on reactive aspects of control by comparing activity between i and n_i_ trial types within I blocks. Brain regions demonstrating trial-related Stroop interference may represent the neural underpinnings of biasing attention towards task-relevant dimensions of the particular stimulus (i.e. the specific ink color), detection and resolution of interference, selection of task-relevant responses, inhibition of irrelevant prepotent responses, and/or evaluation of one's response [57]. Importantly, these mechanisms are all implemented *following* the appearance of the stimulus and are thus appropriately described as “reactive control” mechanisms according to the Dual Mechanisms of Control model [10]. Mid-dLPFC (near BA 9/46) is thought to bias attention toward task-relevant representations (i.e. blue as opposed to yellow color) [9], whereas medial prefrontal regions near ACC and SMA/pre-SMA are thought to implement late-stage and response-related aspects of control, including selection of the task-relevant response [57], [58]. Thus, if adolescents rely more heavily on “ad-hoc” reactive control mechanisms, they might exhibit increased transient activity in medial PFC and/or mid-dLPFC regions compared to adults.

**Figure 3:**
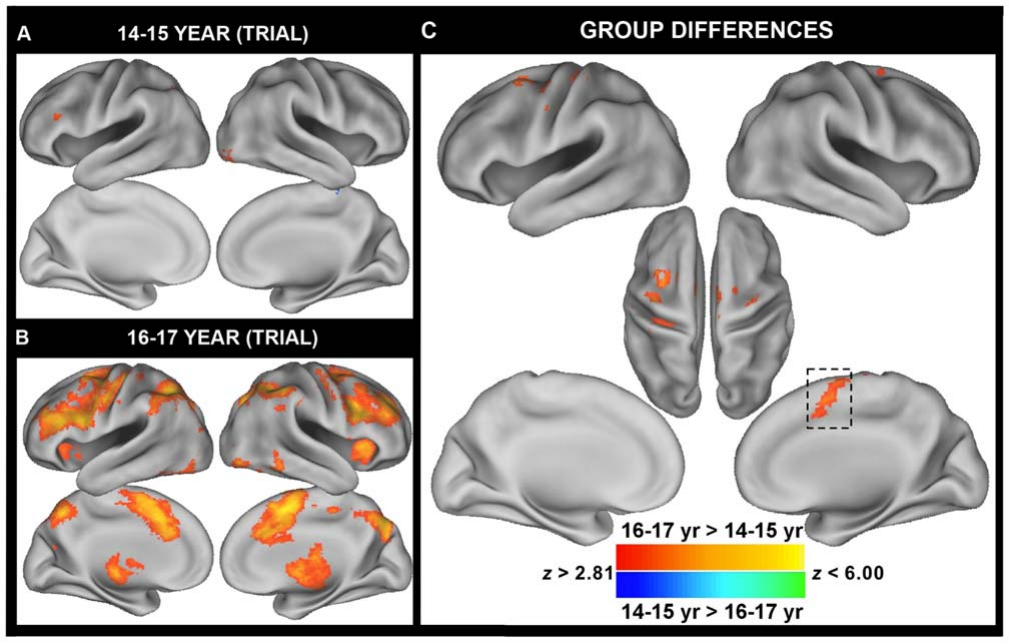
Between-group differences in trial-related Stroop activity reveal heterogeneity within the adolescent group. Trial-related (transient) Stroop contrasts were examined by comparing incongruent (i) and neutral (n_i_) trials within incongruent blocks. Since no differences in activity were observed between adults and adolescents, adolescents were subsequently divided into two groups of **A.** 15 14–15 year olds and **B.** 17 16–17 year olds. Voxels in red indicate greater activity on i trials compared to n_i_ trials and voxels in blue indicate greater activity on n_i_ trials compared to i trials. **C.** Between-group differences revealed older adolescents exhibited elevated BOLD activity in a number of response-related regions including pre-SMA, ACC, lateral pre-motor, motor, and somatosensory areas. *Note:* A voxel-wise threshold of *p*<0.005 and a cluster-wise threshold of >103 contiguous voxels were applied to the statistical maps using Monte Carlo permutation simulations (AlphaSim). Results are projected onto a surface template (Caret Software) [83].

**Figure 4:**
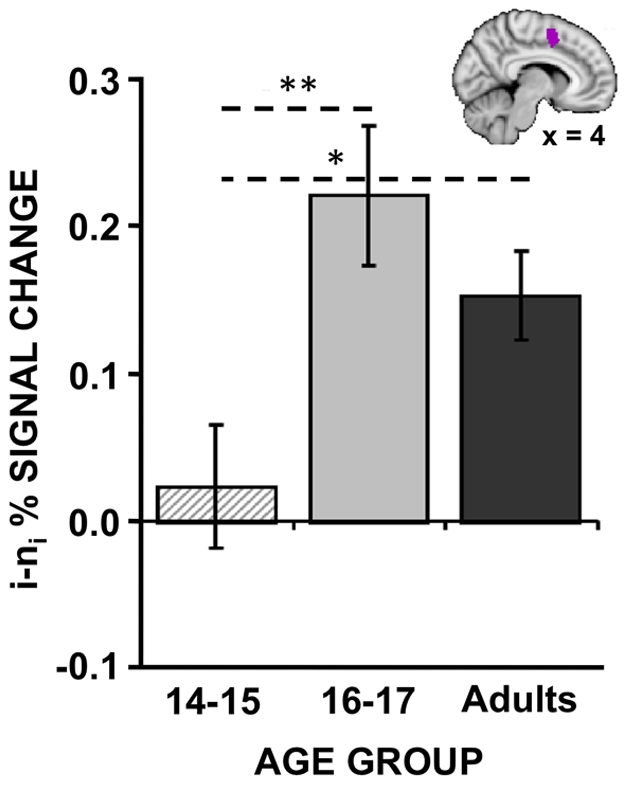
Trial-related group differences in the ACC/pre-SMA. Percent signal change for the contrast between incongruent (i) and neutral (n_i_) trials within incongruent blocks was extracted from the anterior cingulate cortex (ACC)/ pre-supplementary motor area (pre-SMA) cluster shown in a sagittal slice [see box in **Figure 3**]. Activity within this region is plotted separately for young adolescents (14–15 year olds), older adolescents (16–17 year olds), and adults (18–25 year olds). Older adolescents activated the midline cluster significantly more than younger adolescents and numerically (but non-significantly) more than adults. *Note:* *p<0.05; **p<0.01. Error bars reflect standard error of the mean.

To our surprise, we did not observe any significant differences between adults and adolescents for the event-related contrast of i vs. n_i_ trials within I blocks. Furthermore, only two small clusters in the right inferior frontal gyrus/frontal pole (x = 46, y = 32, = 6) and the right parietal operculum (x = 46, y = −24, z = 20) exhibited linear relationships with age. However, post-hoc exploration revealed that the BOLD pattern elicited by the adolescent group was strongly dependent upon age *within* the adolescent group. When the adolescent group was subdivided into a group of 15 participants between the ages of 14–15 and 17 participants between the ages of 16–17 to examine if reactive control processes develop earlier in adolescence, significant differences between the two adolescent age groups were observed. Whereas the 16–17 year olds robustly activated a number of prefrontal and parietal regions typically involved in cognitive control and response inhibition or monitoring of attention (**Figure 3B**), the 14–15 year olds minimally activated these regions (**Figure 3A**). A direct comparison revealed significantly increased activity in 16–17 year olds, relative to 14–15 year olds, notably within ACC/pre-SMA as well as other response-related regions including premotor and primary motor cortex (**Figure 3C** **;** **Table 4**). Interestingly, when the percent signal change within the ACC/pre-SMA region was extracted for all participants, the 16–17 year olds exhibited numerically greater activity even compared to adults, although this difference was not significant (**Figure 4**; 16–17 year olds: 0.22%, Adults: 0.15%, two-tailed t-test: *t*(48) = 1.26, *p* = 0.21).

**Table 4:** Trial-related fMRI group differences.

	# Voxels	x	y	z	Peak *z*-statistic		
					Diff	Adults	Adol
**Inc – Neut Trials (16–17 yr>14–15 yr)**							
Precentral Gyrus/White Matter (L) *λ*		−28	−10	38	3.86	3.64	−1.76
SMA/Paracingulate Gyrus/ACC (R) *λ*		4	4	50	3.75	5.64	0.47
Precentral Gyrus/MFG/SFG (L) *λ*		−36	−8	56	3.75	4.55	−0.61
Precentral Gyrus (R)	190	16	−22	56	3.65	3.24	−1.81
Lateral Occipital Cortex (R)	152	32	−60	28	3.89	5.36	−0.07
Precentral Gyrus/Postcentral Gyrus (L)	156	−40	−28	64	3.21	3.30	−1.18

*Note:* See Methods for details concerning region identification. No regions exhibited significant group differences between adolescents and adults. No regions exhibited significantly greater activity in 14–15 yr olds compared to 16–17 yr olds.

ACC = Anterior Cingulate Cortex; MFG = Middle Frontal Gyrus; SFG = Superior Frontal Gyrus; SMA = Supplementary Motor Area.

*λ* = regions part of a single cluster of 1459 voxels.

### Trade-offs between Blocked and Trial-Related Activity?

One question that arises is whether the two adolescent groups exhibited trade-offs in their use of reactive and proactive control mechanisms, consistent with an “either-or” approach to increasing task demands. In order to explore this possibility and to mirror analyses done with respect to trial-related activity, we directly compared the two adolescent groups with respect to their blocked activity. Whereas the older adolescents exhibited more trial-related (i.e. reactive) activity in late-stage response-related regions than younger adolescents, the two adolescent groups did not differ significantly in terms of their blocked-related activity (I-N blocks), both when analyzed in a whole-brain manner (voxel-wise threshold of *p*<0.005 and a cluster-wise threshold of >103 contiguous voxels) or when restricting our analyses to the IFJ/pDLPFC ROI (**Figure 2B**; 14–15 year old I-N % signal change = 0.09; 16–17 year old I-N % signal change = 0.15; *p*-value from group t-test = 0.28). However, within this ROI, both adolescent groups exhibited significantly reduced blocked activity compared to adults (Adults vs. 16–17 year olds, *p* = 0.018; Adults vs. 14–15 year olds, *p*<0.001).

These results are consistent with the idea that proactive and reactive control exhibit different developmental trajectories. In particular, our results suggest that compared to adults, the 14–15 year olds seem to show reduced BOLD activity associated with both proactive and reactive control. However, the 16–17 year olds only exhibit reductions in BOLD activity linked to proactive control. In these older adolescents, activity linked to reactive control is elevated, consistent with a compensatory reliance on reactive response-related mechanisms. Consistent with this observation, the younger adolescents, who did not employ this potential compensatory reactive mechanism, trended towards more errors than the older adolescents (proportion accuracy interference: 14–15 year olds = 5.22%, 16–17 year olds = 2.52%, two-tailed *t*-test: *t*(28) = −1.90; *p* = 0.068).

### Relationship between fMRI Activity, Stroop Performance, and Self-Report Measures of Cognitive/Social Control

As described above, adolescents exhibited reduced blocked activity in IFJ/pDLPFC compared to adults during the Stroop task. Thus, these results are consistent with the hypothesis that adolescents may experience difficulty implementing and/or maintaining task goals over extended durations. A logical alternative possibility, however, is that adolescents are more functionally efficient given they required less brain activity to achieve similar levels of performance. To rule out this alternative explanation, we examined relationships between brain activity and both Stroop performance and real-world behaviors.

#### fMRI Activity and Stroop Performance

For each participant, we extracted fMRI activity (i.e. I-N blocked percent signal change) in the IFJ/pDLPFC since this region exhibited consistent group differences across the blocked contrasts (see **Figure 1** and **Figure S1**) and is a key component of our Cascade-of-Control model [5]. Then for each group separately, we performed a partial correlation with age as a covariate of non-interest between IFJ/pDLPFC activity (I-N blocks) and performance (i.e. percentage increase in RT interference as well as percentage increase in accuracy interference). Though not statistically significant, the relationship in adolescents was in the expected direction. Increased IFJ/pDLPFC blocked activity (I-N blocks) was associated with better Stroop performance (pDLPFC/IFJ I-N percent signal change×proportion blocked RT Stroop interference: partial *r* = −0.22, *p* = 0.25; I-N percent signal change×Accuracy measures: partial *r* = 0.23, *p* = 0.22). In other words, the adolescents who activated their IFJ/pDLPFC more on I compared to N blocks were faster to respond and made fewer errors on I blocks than those who didn't activate this region as strongly. Considering structural studies indicating the dLPFC and inferior frontal sulcus develop last among the frontal regions [i.e. 59]–[61], these results suggest that reduced blocked IFJ/pDLPFC activity in adolescents is indicative of under-developed proactive control mechanisms.

In contrast, adults exhibited the opposite relationship with Stroop behavior, such that adults who activated their IFJ/pDLPFC to a lesser degree trended toward more successful Stroop behavior (IFJ/pDLPFC I-N percent signal change×proportion blocked RT Stroop interference: partial *r* = 0.33, *p* = 0.07; I-N percent signal change×Accuracy measures: partial *r* = −0.17, *p* = 0.35). Moreover, these relationships were significantly different than those observed in adolescents (group differences in correlations with proportion RT interference: *z* = −2.16, *p* = 0.031; group differences in correlations with proportion accuracy interference: *z* = 1.54, *p* = 0.12). These results are consistent with the idea that in adults, decreased pDLPFC/IFJ activity might reflect improved efficiency of proactive control, possibly due to more developed prefrontal mechanisms and more experience utilizing proactive control.

#### fMRI Activity and Self-Report Measures of Cognitive/Social Control

If blocked recruitment of IFJ/pDLPFC indexes proactive maintenance of task goals, then increased neural engagement of these regions in adolescents might be beneficial for adolescents' everyday cognitive and social behavior including planning, control of one's impulses, and resistance to peer pressure. To explore this possibility, we created a composite measure of cognitive/social control by averaging the z-scores from participants' self-report questionnaires pertaining to impulse control, planning ahead, and resistance to peer influence (see **Materials and Methods**). Higher scores are indicative of a better perceived ability to control one's own behavior and plan ahead. Adults trended toward reporting higher cognitive/social control than adolescents (two-tailed *t*-test: *t*(56) = 1.71, *p* = 0.093).

**Figure 5:**
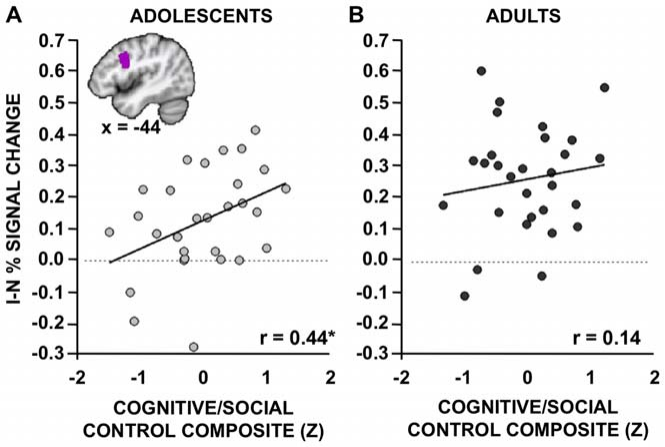
Relationships between blocked Stroop activity and self-report measures of cognitive/social control. Percent signal change reflecting the contrast of Incongruent (I) vs. Neutral (N) blocks was extracted from the IFJ/pDLPFC seed that exhibited group differences in Figure 1C (see box in **Figure 1C** and inset in current figure). Activity within this region was correlated with self-report measures of cognitive/social control separately for **A.** adolescents and **B.** adults. The cognitive/social control composite represents participants' average z-scores from individual questionnaires assessing impulse control, planning, and resistance to peer pressure. **A.** In adolescents, a significant positive relationship between the two variables was observed such that adolescents who activate the *a priori* region to a greater degree reported greater cognitive/social control. **B.** The relationship in adults, although positive, was not significant. *Note:* *p<0.05.

Both *a priori* ROI and whole-brain exploratory approaches were implemented to to explore whether relationships within our *a priori* regions remained significant at more conservative thresholds, and to consider the possibility that regions other than those predicted might exhibit similar brain-behavior relationships. Correlations were performed between measures of fMRI activity within the *a priori* IFJ/pDLPFC ROI and composite measures of cognitive/social control, separately for adolescents and adults. Consistent with the above predictions, a significant positive relationship between IFJ/pDLPFC blocked I-N Stroop activity and cognitive/social control was observed in adolescents (**Fig. 5A**; *r*(26) = 0.44, *p* = 0.020), even when controlling for the effect of age on both variables (partial *r*(25) = 0.45, *p* = 0.020). In other words, adolescents who activate their pDLPFC to a greater degree report being better able to control their behaviors and plan ahead. A much weaker, non-significant relationship between the two variables was observed in adults (**Figure 5B**: *r*(27) = 0.14, *p* = 0.47; partial *r*(26) = 0.13, *p* = 0.50). However, the correlations were not significantly different between the two age groups after Fisher's z transformation (*z* = 1.26, one-tailed *p* = 0.10).

To examine whether blocked Stroop activity (I-N activity) in regions other than the IFJ/pDLPFC exhibit significant relationships with cognitive/social control, we performed whole-brain regressions separately for adolescents and adults. The cognitive/social control composite score for each individual, along with his/her demeaned age, were included in the GLM. In addition to pDLPFC, activity in the frontal pole and bilateral inferior frontal gyrus extending into the orbitofrontal cortex predicted better cognitive/social control in adolescents (**Figure S2A; Table S2**). In adults, greater activity in a single cluster in the left medial PFC near BA8/9 predicted better cognitive/social control (**Figure S2B**; **Table S2**).

## Discussion

The present study employed a hybrid blocked/event-related fMRI Stroop paradigm and self-report measures of cognitive/social control to investigate developmental trajectories of reactive and proactive mechanisms of cognitive control across adolescence and early adulthood. Results suggest early functional development of reactive, response-related aspects of control followed by later development of proactive, sustained aspects of control that may become more efficient through early adulthood. Collectively, these results underscore the importance of considering cognitive control as a heterogeneous construct and reveal that adolescence marks an important neurobiological transition period facilitating the implementation and extended maintenance of task goals.

### Delayed maturation of proactive goal maintenance

Blocked fMRI Stroop analyses revealed that compared to adults, adolescents under-activated a network of frontal-parietal brain regions thought to play a role in cognitive control and attention. Group differences were consistently present in a posterior lateral PFC region near the junction of BA 8, 6, and 44 [according to the Brodmann maps of 62]. Although the literature has yet to converge on a precise naming convention for the posterior lateral PFC region, it comprises an area that has sometimes been referred to as inferior frontal junction [for review see 63] and extends dorsally into the posterior dLPFC [5], [63]. This large region spanning multiple anatomical boundaries is robustly activated across a number of tasks requiring the implementation and maintenance of task sets, including the Stroop task [8], [63]–[67], task-switching paradigms [66], [68], and other tasks requiring the representation and/or maintenance of abstract rules [69]–[71], [ see Figure 4B of 72]. Additionally, the superior part of this region (pDLPFC) represents a key player in the Cascade-of-Control Model, biasing attention towards task-relevant representations in an anticipatory “proactive” manner [5]–[7]. Since participants performed the same task (color identification) across blocks of trials, activity sustained over the course of the block might index top-down anticipatory control (but see caveat in methods). Thus, our results are consistent with the possibility that adolescents are poorer at sustaining task goals over extended periods of time.

Consistent with this conclusion, adolescents who exhibited greater blocked IFJ/pDLPFC activity exhibited better self-report composite measures of impulse control, planning ability, and resistance to peer influence. Additionally, though the relationship was non-significant, adolescents with increased blocked IFJ/pDLPFC activity also performed better on the Stroop task. Thus, all the findings point in the same direction, namely that increased prefrontal activity during adolescence is associated with improved cognitive control.

Although the associations we observed indicate nothing about causation, adolescence marks a period of important ongoing structural changes. Prefrontal and parietal gray and white matter continue to develop through adolescence, with the dLPFC being particularly delayed [59]–[61], [73]–[75], [ reviewed in 2], [27]–[34]. Furthermore, functional connectivity between brain regions develops from a “local to distributed” pattern across adolescence [76], [ see 77 for review]. Thus, one likely possibility is that these changes in brain development underlie the observed maturation of cognitive control. Alternatively, it could be that increasing age or an increased tendency towards self-control results in more practice with such control, which in turn helps to sculpt the structure and function of prefrontal regions involved in cognitive control. Obviously, these two possibilities need not be mutually exclusive.

Although between-group comparisons revealed adolescents significantly under-activated the lateral prefrontal cortex compared to adults, the relationship between prefrontal activity and age was curvilinear, peaking approximately at age 21 and decreasing thereafter. Thus, considerable heterogeneity in IFJ/pDLPFC activity was present even in the adult group, and this heterogeneity was supported by weaker relationships between IFJ/pDLPFC activity and self-report measures of cognitive/social control in adults. What might give rise to the inverted J-shaped relationship between prefrontal activity and age? Prior studies have found significant environmental and lifestyle-induced influences on neural development and cognitive function [e.g. 78]. Given that early adulthood marks a period of substantial lifestyle change marked by academic, occupational, financial, and social responsibilities that benefit from foresight and planning [79], [80], our results may provide additional support for the idea that adolescence and emerging adulthood are shaped by a combination of ongoing prefrontal development and lifestyle-induced functional plasticity.

### Early maturation of reactive, response-related aspects of control

Although improved behavioral performance on the Stroop task can be accomplished by sustained, anticipatory mechanisms of control, cognitive control can also be implemented transiently on a “when-needed” basis particularly when faced with the more difficult incongruent trials. The Cascade-of-Control model posits that such late-stage transient aspects of control are implemented by the ACC to select and evaluate task-relevant responses [5]. Importantly, the degree to which one successfully implements early or sustained proactive aspects of control influences the degree to which later stages of control are needed to yield successful behavior [7], [81].

Although no differences in activation were observed between adults and adolescents for the ACC, heterogeneous patterns of ACC/pre-SMA activation within the adolescent group suggest a compensatory reactive response (**Figure 3**). Older adolescents (age 16–17) sustained somewhat low levels of lateral PFC but recruited late-stage reactive, response-related mechanisms of control subserved largely by ACC/pre-SMA. These late-stage mechanisms were not recruited as strongly by the younger adolescents. As younger adolescents (age 14–15) made more errors than older adolescents (age 16–17), ACC/pre-SMA recruitment in adolescents may be beneficial for successful cognitive control.

### Relation to prior developmental fMRI studies

Our results are broadly consistent with prior developmental fMRI studies, particularly those that demonstrate reduced prefrontal activity in adolescents compared to adults ([e.g. 14]–[24], [26], [ reviewed in 2], [27], [28], [30] as well as those that suggest different developmental trajectories of lateral and medial PFC function [16], [24]. However, it should be noted that the comparison between studies is complicated by the use of different tasks, type of demand for cognitive control, sample sizes, and fMRI designs and analysis methods (e.g. blocked vs. event-related).

The present study extends many of these prior findings by implementing a hybrid blocked/event-related design, allowing us to examine both proactive and reactive component processes of control in the same experimental task and the same group of participants (but see caveat in **Materials and Methods**). Of note, Velanova and colleagues [16] took a somewhat similar approach using a state-item design by examining the development of sustained and transient activity during an occulomotor inhibiton task in a large sample of children (age 8–12), adolescents (13–17) and adults (18–27). Similar to the present results, Velanova and colleagues found that a region near the IFJ (although right-lateralized in their study) that exhibited greater sustained activity in adults than adolescents and children. Thus, our study provides additional evidence of involvement of the IFJ/pDLPFC in adults compared to adolescents, not only as previously observed in a relatively low-level occulomotor task requiring inhibition of motor responses [16], but also in a more higher-level task (i.e. the Stroop task) for which response inhibition is but one process required for successful performance. Additionally, our study extends the interesting findings of Velanova and colleagues by demonstrating that blocked-related activity within the IFJ/pDLPFC predicts real-world adolescent behaviors including impulse control, planning behavior and resistance to peer influence.

### Potential Limitations

The results of the present study are limited in that they do not examine cognitive control in early adolescence. Research suggests that the onset of adolescence is a gradual process that may begin earlier than age 14, likely coinciding with puberty [e.g. 82], and as such our data do not speak to other changes in the neural bases in cognitive control that may occur at earlier ages. Additionally, as noted in the **Materials and Methods**, the present study explored blocked and event-related effects using separate GLMs, similar to our prior studies [38], [39]. Though the individual trial analyses are considered pure estimates of transient differences between i and n_i_ trials, the blocked analyses may not be considered pure estimates of sustained activity because it is possible they may be confounded by transient effects from individual trials within the block. We think that the blocked effects observed in our study are unlikely to be driven by trial-related activity because none of the regions that exhibited group differences in blocked activity also exhibited group differences in trial-related activity (see **Materials and Methods** for additional discussion). Nevertheless, given this potential limitation, the blocked effects in the present study should be interpreted with caution.

### Conclusion

In summary, the present findings suggest that adolescence and emerging adulthood reflect important developmental transition periods marked by relatively early engagement of neural systems for reactive, response-related aspects of control followed by later engagement of neutral systems for anticipatory, proactive control. Importantly, these differential neurobiological profiles of cognitive control might partly account for individual differences in stereotypical adolescent behaviors, including impulsivity and lack of foresight. As previous studies suggest that strategy use in young and older adults may be malleable [12], future studies should investigate whether adolescents' use of proactive control strategies could be altered by targeted training procedures. In addition, since the present study is cross-sectional in nature, future studies should examine causal roles of prefrontal activity, as well as anatomy and functional connectivity, on adolescent behavior using longitudinal designs [25].

## Supporting Information

Figure S1 Between-group blocked differences in the three Stroop task conditions compared to fixation. Clusters of BOLD activity demonstrating significant between-group differences are displayed for the contrasts: **A.** Incongruent blocks (I) compared to fixation, **B.** Congruent blocks (C) compared to fixation, and **C.** Neutral blocks (N) compared to fixation. Red voxels indicate greater activity in adults compared to adolescents; blue voxels indicate greater activity in adolescents compared to adults. For all three contrasts, adults activated left lateral prefrontal regions (mid- and posterior- dorsolateral prefrontal cortex/ inferior frontal junction) to a greater degree than adolescents. *Note:* A voxel-wise threshold of *p*<0.005 and a cluster-wise threshold of >103 contiguous voxels have been applied to the statistical maps using Monte Carlo permutation simulations (AlphaSim). Results are projected onto a surface template (Caret Software).
(TIF)

Figure S2 Whole-brain exploratory relationships between fMRI blocked Stroop activity and self-report measures of cognitive/social control. Whole-brain correlation analyses between the contrast of I-N blocks and individual self-report measures of cognitive/social control were performed separately for adolescents and adults, including and age as a covariate of non-interest. **A.** In adolescents, activity in several regions was significantly positively correlated with self-report measures of cognitive/social control: **a**) a cluster at or near left posterior dLPFC (pDLPFC) overlaping with the *a priori* region (see **Fig. 5**), and **b**) bilateral clusters near inferior frontal gyrus extending into the orbitofrontal cortex and frontal pole. **B.** In adults, activity in a left medial PFC region near BA8/9 positively correlated with self-report measures of cognitive/social control. *Note:* A voxel-wise threshold of *p*<0.005 and a cluster-wise threshold of >103 contiguous voxels were applied to the statistical maps as calculated by Monte Carlo permutation simulations (AlphaSim). Results are projected onto a surface template (Caret Software).
(TIF)

Table S1 fMRI group differences for task blocks compared to fixation.
(DOC)

Table S2 Relationships between blocked fMRI stroop activity (I-N) and self-report measures of cognitive/social control.
(DOC)
