# Supplementary figures and images for: Cognitive Control in Adolescence: Neural Underpinnings and Relation to Self-Report Behaviors
Source: PLoS One. 2011 Jun 28;6(6):e21598. doi: 10.1371/journal.pone.0021598 (PMC3125248; doi:10.1371/journal.pone.0021598)

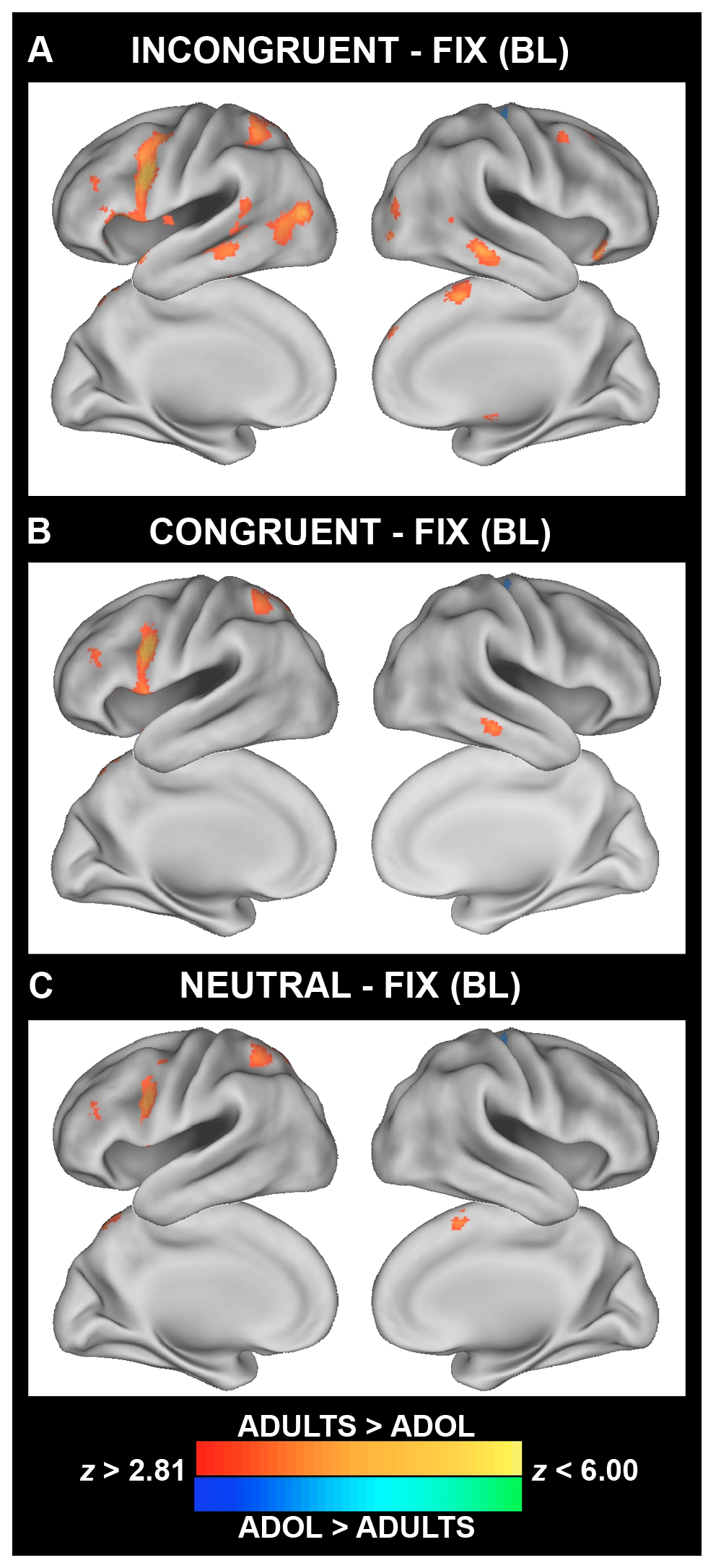

Supplement: Figure S1 — Between-group blocked differences in the three Stroop task conditions compared to fixation. Clusters of BOLD activity demonstrating significant between-group differences are displayed for the contrasts: A. Incongruent blocks (I) compared to fixation, B. Congruent blocks (C) compared to fixation, and C. Neutral blocks (N) compared to fixation. Red voxels indicate greater activity in adults compared to adolescents; blue voxels indicate greater activity in adolescents compared to adults. For all three contrasts, adults activated left lateral prefrontal regions (mid- and posterior- dorsolateral prefrontal cortex/ inferior frontal junction) to a greater degree than adolescents. Note: A voxel-wise threshold of p<0.005 and a cluster-wise threshold of >103 contiguous voxels have been applied to the statistical maps using Monte Carlo permutation simulations (AlphaSim). Results are projected onto a surface template (Caret Software). (TIF) [file pone.0021598.s001.tif]

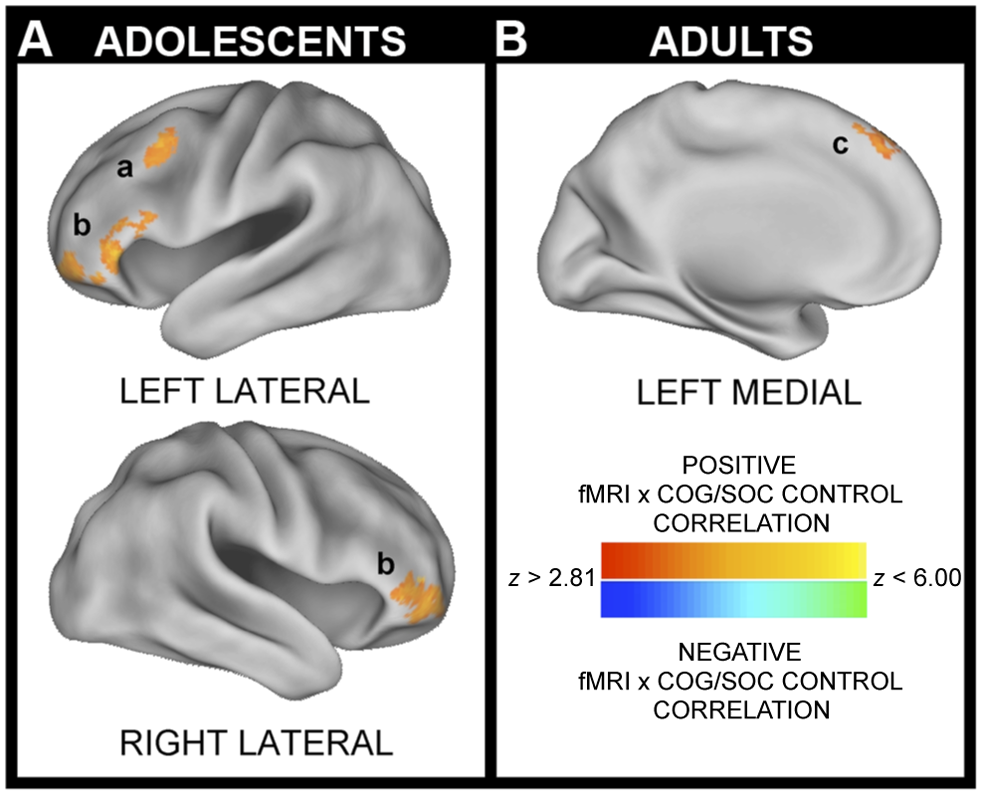

Supplement: Figure S2 — Whole-brain exploratory relationships between fMRI blocked Stroop activity and self-report measures of cognitive/social control. Whole-brain correlation analyses between the contrast of I-N blocks and individual self-report measures of cognitive/social control were performed separately for adolescents and adults, including and age as a covariate of non-interest. A. In adolescents, activity in several regions was significantly positively correlated with self-report measures of cognitive/social control: a) a cluster at or near left posterior dLPFC (pDLPFC) overlaping with the a priori region (see Fig. 5 ), and b) bilateral clusters near inferior frontal gyrus extending into the orbitofrontal cortex and frontal pole. B. In adults, activity in a left medial PFC region near BA8/9 positively correlated with self-report measures of cognitive/social control. Note: A voxel-wise threshold of p<0.005 and a cluster-wise threshold of >103 contiguous voxels were applied to the statistical maps as calculated by Monte Carlo permutation simulations (AlphaSim). Results are projected onto a surface template (Caret Software). (TIF) [file pone.0021598.s002.tif]
