# Supplementary material for: Cognitive Control in Adolescence: Neural Underpinnings and Relation to Self-Report Behaviors
Source: PLoS One. 2011 Jun 28;6(6):e21598. doi: 10.1371/journal.pone.0021598 (PMC3125248; doi:10.1371/journal.pone.0021598)
Supplement: Table S1 — fMRI group differences for task blocks compared to fixation. (DOC) [file pone.0021598.s003.doc]

**Table S1:** fMRI Group Differences for Task Blocks Compared to Fixation

|  | # Voxels | x | y | z | Peak *z*-statistic | | |
| --- | --- | --- | --- | --- | --- | --- | --- |
|  |  |  |  |  | Diff | Adults | Adol |
| **Incongruent Blocks - Fixation Blocks** |  |  |  |  |  |  |  |
| *Adults > Adolescents* |  |  |  |  |  |  |  |
| Precentral Gyrus/IFG/MFG (L) *λ* |  | -44 | 4 | 30 | 5.15 | 9.47 | 4.82 |
| IFG/Frontal Operculum (L) *λ* |  | -56 | 20 | -4 | 4.60 | 2.79 | -4.22 |
| Frontal Pole/SFG (R) | 150 | 4 | 60 | 34 | 4.56 | 2.70 | -3.96 |
| OFC/Temporal Pole (R) | 302 | 46 | 22 | -12 | 4.01 | 7.32 | 2.66 |
| SFG/Paracingulate Gyrus (R) | 182 | 6 | 16 | 54 | 3.81 | 7.63 | 2.88 |
| Globus Pallidus (R) | 108 | 14 | 2 | -6 | 3.56 | 6.47 | 2.19 |
| MFG (L) | 198 | -36 | 32 | 24 | 3.50 | 6.56 | 2.46 |
| MFG/SFG (R) | 220 | 30 | 20 | 46 | 3.24 | 1.00 | -3.77 |
| MTG/LOC/Angular Gyrus (R) | 216 | 64 | -60 | 8 | 4.67 | 0.95 | -5.38 |
| LOC/Occipital Pole (R) | 115 | 42 | -88 | 0 | 4.23 | 5.59 | -0.17 |
| LOC/MTG (L) | 524 | -42 | -74 | 14 | 4.06 | -0.68 | -5.93 |
| Lingual Gyrus/Occipital Pole (R) | 143 | 2 | -92 | -20 | 3.94 | 6.05 | 1.18 |
| MTG (R) | 286 | 64 | -36 | -12 | 3.89 | 5.86 | 0.73 |
| Precuneus/LOC (L) | 559 | -2 | -74 | 56 | 3.80 | 5.68 | 0.65 |
| SMG/Angular Gyrus/STG (L) | 180 | -66 | -48 | 22 | 3.52 | 2.20 | -3.11 |
| MTG/ITG (L) | 378 | -64 | -38 | -12 | 3.49 | 4.98 | 0.14 |
| *Adolescents > Adults* |  |  |  |  |  |  |  |
| Precentral Gyrus/ Postcentral Gyrus (R) | 164 | 26 | -22 | 60 | 3.38 | -1.75 | 2.80 |
| **Congruent Blocks - Fixation Blocks** |  |  |  |  |  |  |  |
| *Adults > Adolescents* |  |  |  |  |  |  |  |
| Precentral Gyrus/IFG/MFG (L) *Ω* |  | -44 | 6 | 24 | 4.94 | 2.62 | 7.56 |
| OFC/IFG/Temporal Pole (L) *Ω* |  | -54 | 20 | -8 | 4.10 | 2.89 | -3.41 |
| MFG (L) *Ω* |  | -34 | 32 | 24 | 3.68 | 6.40 | 1.43 |
| Precuneus/LOC/SPL (L) | 443 | -2 | -74 | 58 | 3.46 | 4.51 | -0.37 |
| ITG/MTG (R) | 114 | 60 | -28 | -18 | 3.44 | 4.40 | -0.49 |
| *Adolescents > Adults* |  |  |  |  |  |  |  |
| Precentral Gyrus/Postcentral Gyrus (R) | 114 | 28 | -24 | 62 | 3.41 | -2.87 | 2.11 |
| **Neutral Blocks - Fixation Blocks** |  |  |  |  |  |  |  |
| *Adults > Adolescents* |  |  |  |  |  |  |  |
| Precentral Gyrus/IFG/MFG (L) | 552 | -44 | 4 | 22 | 4.37 | 6.58 | 0.96 |
| MFG (L) | 248 | -34 | 32 | 22 | 3.83 | 5.89 | 0.86 |
| SFG/Supplementary Motor Area (R) | 114 | 2 | 14 | 64 | 3.82 | 2.50 | -3.33 |
| Caudate (L) | 147 | -10 | -2 | 14 | 3.21 | 4.85 | 0.43 |
| Precuneus/LOC/SPL (L) | 610 | -2 | -74 | 58 | 4.15 | 4.57 | -1.46 |
| *Adolescents > Adults* |  |  |  |  |  |  |  |
| Precentral Gyrus/Postcentral Gyrus (R) | 119 | 28 | -24 | 62 | 3.51 | -2.42 | 2.77 |

*Note:* See Methods for details concerning region identification. IFG = Inferior Frontal Gyrus; ITG = Inferior Temporal Gyrus; LOC = Lateral Occipital Cortex**;** MFG = Middle Frontal Gyrus; MTG = Middle Temporal Gyrus; OFC = Orbitofrontal Cortex; SFG = Superior Frontal Gyrus; SMG = Supramarginal Gyrus; SPL = Superior Parietal Lobule; STG = Superior Temporal Gyrus; *λ =* regions part of a single cluster of 1798 voxels

*Ω =* regions part of a single cluster of 1361 voxels
