# Supplementary material for: Cognitive Control in Adolescence: Neural Underpinnings and Relation to Self-Report Behaviors
Source: PLoS One. 2011 Jun 28;6(6):e21598. doi: 10.1371/journal.pone.0021598 (PMC3125248; doi:10.1371/journal.pone.0021598)
Supplement: Table S2 — Relationships between blocked fMRI stroop activity (I-N) and self-report measures of cognitive/social control. (DOC) [file pone.0021598.s004.doc]

**Table S2:** Relationships between Blocked fMRI Stroop Activity (I-N) and Self-Report Measures of Cognitive/Social Control

|  | # Voxels | x | y | z | Peak *z*-statistic |
| --- | --- | --- | --- | --- | --- |
| *Adolescents* |  |  |  |  |  |
| IFG/OFC/Frontal Pole (L) *λ* |  | -46 | 32 | 0 | 3.92 |
| Frontal Pole (L) *λ* |  | -36 | 58 | -8 | 3.56 |
| Frontal Pole (R) | 560 | 38 | 50 | -2 | 3.90 |
| Middle Frontal Gyrus (L) | 158 | -38 | 10 | 42 | 3.55 |
| *Adults* |  |  |  |  |  |
| Superior Frontal Gyrus/Frontal Pole (L) | 165 | -10 | 40 | 44 | 3.35 |

*Note:* See Methods for details concerning region identification. IFG = Inferior Frontal Gyrus; OFC = Orbitofrontal Cortex

*λ =* regions part of a single cluster of 731 voxels
